# *Bacillus subtilis* MreB Orthologs Self-Organize into Filamentous Structures underneath the Cell Membrane in a Heterologous Cell System

**DOI:** 10.1371/journal.pone.0027035

**Published:** 2011-11-01

**Authors:** Felix Dempwolff, Christian Reimold, Michael Reth, Peter L. Graumann

**Affiliations:** 1 Mikrobiologie, Fakultät für Biologie, Universität Freiburg, Freiburg, Germany; 2 Immunbiologie, Fakultät für Biologie, Universität Freiburg, Freiburg, Germany; 3 Bioss, Universität Freiburg, Freiburg, Germany; University of Colorado, Boulder, United States of America

## Abstract

Actin-like bacterial cytoskeletal element MreB has been shown to be essential for the maintenance of rod cell shape in many bacteria. MreB forms rapidly remodelling helical filaments underneath the cell membrane in *Bacillus subtilis* and in other bacterial cells, and co-localizes with its two paralogs, Mbl and MreBH. We show that MreB localizes as dynamic bundles of filaments underneath the cell membrane in *Drosophila* S2 Schneider cells, which become highly stable when the ATPase motif in MreB is modified. In agreement with ATP-dependent filament formation, the depletion of ATP in the cells lead to rapid dissociation of MreB filaments. Extended induction of MreB resulted in the formation of membrane protrusions, showing that like actin, MreB can exert force against the cell membrane. Mbl also formed membrane associated filaments, while MreBH formed filaments within the cytosol. When co-expressed, MreB, Mbl and MreBH built up mixed filaments underneath the cell membrane. Membrane protein RodZ localized to endosomes in S2 cells, but localized to the cell membrane when co-expressed with Mbl, showing that bacterial MreB/Mbl structures can recruit a protein to the cell membrane. Thus, MreB paralogs form a self-organizing and dynamic filamentous scaffold underneath the membrane that is able to recruit other proteins to the cell surface.

## Introduction

Prokaryotes show an amazing variety of different cell shapes, as do many eukaryotic single cell organisms. The maintenance of rod shape is essential for many bacteria, and is based on an actin-like MreB cytoskeleton. MreB is in most cases essential for viability and its depletion leads to a loss of rod cell shape, leaving the cells as large round spheres that eventually lyse. *In vitro*, MreB forms double filaments very similar to those formed by actin (except that actin filaments are helical), but also sheets of filaments [1]. *In vivo*, MreB localizes as helical filaments underneath the cell membrane [2], [3], [4], [5], [6], [7], which in *Bacillus subtilis* and in *Caulobacter crescentus* are highly dynamic and appear to extend at one end and retract at the other end (and thus appear to move along helical tracks) by a ratchet like mechanism [8], [9]. Their dynamic remodelling is important for the function in cell wall maintenance [10]. There is growing evidence that MreB affects cell shape through the positioning of cell wall synthetic enzymes in a helical pattern along the lateral cell membrane [5], [11], [12]. However, the loss of MreB in *B. subtilis* can be compensated by the addition of high concentrations of magnesium and sucrose to the medium [13], indicating that MreB may also play a mechanical function in bacterial cells by stabilizing the cell membrane. A key question for understanding the mode of action of MreB is how the filaments obtain their localization underneath the cell membrane. *B. subtilis* possesses three actin paralogs, MreB, Mbl and MreBH, which colocalize and interact with each other [10], [14]. MreBs can be fused to fluorescent proteins without a loss of function. We wished to obtain knowledge on the architecture of MreB filaments in cells with a wide diameter (more than 1 µm as in bacteria) and to test if this class of proteins may be useable to rationally design 3D structures in heterologous cell systems. We therefore employed eukaryotic S2 cells obtained from *Drosophila* insects and expressed fusions of MreB and/or of Mbl or MreBH to different fluorescent proteins. This system is far diverged from a bacterium, and can take up plasmids to express the encoded proteins after induction of transcription with copper [15]. MreB and actin share only 14% sequence identity, which mostly comprises the conserved ATP binding pocket; the surfaces of MreB and actin do not share any significant sequence similarity [16], such that specific interactions of MreB with actin-interacting proteins are unlikely.

## Results and Discussion

### MreB forms membrane-associated filamentous structures in a heterologous cell system

**Figure 1:**
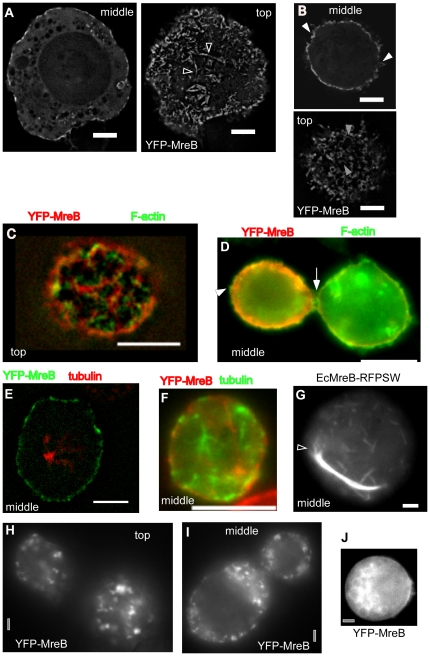
Expression of YFP-MreB or mutant versions in S2 cells. A) Wild type YFP-MreB filaments, shown are a middle plane and top plane of a Z-stack. Triangles indicate bundles of filaments from which a single filament (or thin bundle of filaments) emanates. B) Middle and top planes of a 3D deconvoluted Z-stack of a cell expressing wild type YFP-MreB. C–D) Immunofluorescence of cell expressing YFP-MreB, using phalloidin as stain for actin filaments. Triangles indicate positions of actin filaments that lack any detectable YFP-MreB fluorescence. E–F) Immunofluorescence of cell expressing YFP-MreB, using anti *Drosophila* tubulin antiserum to stain for tubulin filaments. G) *E. coli* MreB (with an internal RFP) expressed in S2 cells, shown is the middle plane. Triangle indicates MreB filaments extending from the end of a filament bundle. H–I) Cells were depleted for ATP by the addition of FCCP, H–I) 20 min after addition, J) 90 min after addition (middle plane is shown). White bars 2 µm (A,B, G) or 5 µm (C–F) respectively, grey bars 2 µm.

When *B. subtilis* MreB was expressed in the heterologous system, a YFP-MreB fusion (that is fully functional in *B. subtilis* cells, [9]) formed up to 7 µm long filaments as soon as 30 min after induction of transcription, all of which were exclusively localized underneath the cell membrane (Fig. 1A and 1B, and movie S1). Therefore, MreB filaments appear to have an intrinsic membrane affinity and do not require a membrane-recruitment factor. This is in contrast to F-actin, which does not have intrinsic membrane affinity.

To rule out that MreB is recruited to the cell membrane by endogenous cytoskeletal elements, we performed immunofluorescence microscopy to visualize MreB in parallel with actin or tubulin. Fig. 1C and 1D show that YFP-MreB did not colocalize with cortical actin filaments. Although both proteins accumulated along the membrane, individual assemblies were not strictly the same, but mostly dissimilar. YFP-MreB did not generally colocalize with tubulin, neither along the membrane, nor within the cytosol (Fig. 1E and 1F), showing that it is not recruited to the membrane by actin or tubulin.

Membrane-associated filament assembly is not a general property of MreB proteins. For example, a functional sandwich (internal fusion) MreB^RFP^ from *Escherichia coli* [17] also formed filamentous structures in S2 cells, but these did not have intrinsic membrane affinity (Fig. 1G and movie S2). In contrast to the MreB filaments in *B. subtilis* cells, which are helical with a diameter of 1.1 µm and have an average pitch of 0.5 µm [9], YFP-MreB filaments in S2 cells were irregularly curved (Fig. 1B) or straight (Fig. 1A). Generally, thin filaments were curved, while thick (bundles of) filaments were straight. Interestingly, we observed a number of arc-like structures, which had a diameter of 500 to 1000 nm (Fig. 1A and B). However, based on the variable structures of MreB filaments, our experiments suggest that the cytoskeletal MreB filaments are not generated through an inherent curvature of MreB filaments *in vivo*. The same observations apply to *E. coli* MreB filaments, which clearly formed bundled structures that eventually ended up at the membrane when their length reached that of the cell diameter (Fig. 1G), but were straight when present within the cytosol away from the membrane.

**Figure 2:**
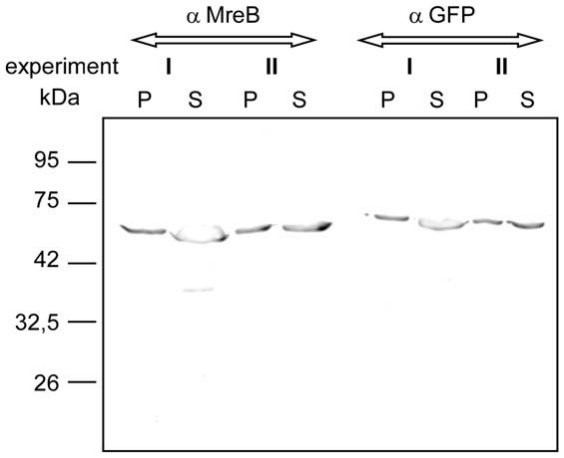
Sedimentation assays. Equal amounts of supernatant (S) or high speed pellet (P) fractions of S2 cells expressing YFP-MreB were loaded onto SDS-PAGE and tested via Western blotting, using anti MreB or anti GFP antibodies. Two independent experiments (each detected by the two different antisera) are shown to illustrate differences in expression levels.

To interpret these results, we determined what percentage of the total MreB proteins were soluble (available for exchange) or insoluble (assembled). We isolated soluble fractions of the cell extract containing non-polymerized MreB, and the high speed centrifugation fraction that would contain polymerized MreB. Equal volumes were loaded onto SDS PAGE and were detected by Western blotting. Fig. 2 shows that at least 50% of MreB was incorporated into filaments (2 different experiments are loaded because transfection rates are somewhat variable, but this does not considerably affect the soluble/assembled ratio). Because we do not know how many MreB filaments are disrupted upon cell lysis, we can therefore state that at least 50% of MreB proteins are within the polymers.

Our experiments clearly demonstrate that MreB filaments can bundle up into larger superstructures. Fig. 1A shows examples of single YFP-MreB filaments (or thin bundles of filaments) that emerge from a much thicker fluorescent structure, and thus from a thicker bundle of filaments. Likewise, EcMreB formed short thin filaments as well as thick and extended bundles of filaments (Fig. 1G), demonstrating that MreB proteins readily form bundles of filaments. EcMreB has also been shown to form straight bundles of filaments in the cytosol of fission yeast cells, which grow by extension at both ends, and show internal remodelling [18]. Thus, filament forming properties of EcMreB are also conserved between different heterologous cell systems.

### MreB filaments are highly dynamic and consist of different fractions

**Figure 3:**
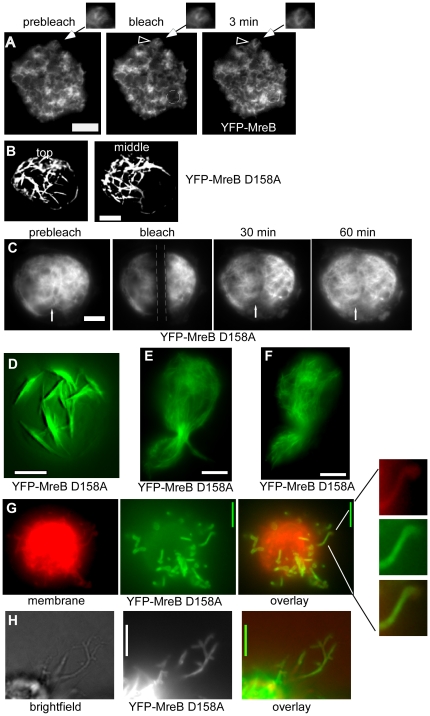
Expression and FRAP experiments of MreB and mutant versions in S2 cells. A) FRAP experiment; an area indicated by a dashed circle is bleached; “min” indicates time after bleaching, clear YFP-MreB filaments can be seen after 3 min. Triangle indicates a filamentous structure that changes within the 3 min interval, which is enlarged in the images above. B) Top plane from a 3D deconvoluted stack of a cell expressing YFP-MreB D158A mutant, C) FRAP experiment of mutant MreB D158A, the stretch indicated by dashed lines is bleached, the white arrow indicates a structure that has recovered after 30 min. D) 3D deconvoluted image of YFP-MreB D158A 5 hours after induction, many of which are still attached to the cell membrane. E) YFP-MreB D158A 12 hours after induction (middle plane), F) YFP-MreB D158A 24 hours after induction (middle plane). G) Top view of a 3D deconvoluted stack of a cell expressing YFP-MreB D158A which forms extrusions that are covered with the cell membrane (one such region is enlarged on the right. Note that the cell membrane is below the focal plane and appears as a haze. H) YFP-MreBD158 protrusions observed by bright field illumination. White bars 2 µm.

**Figure 4:**
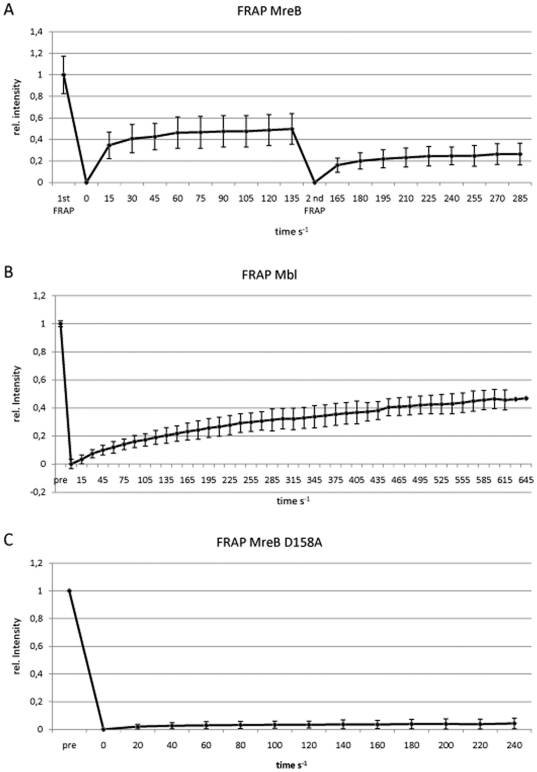
Quantification of FRAP analyses. X-axis show time in seconds, Y-axis relative fluorescence (1 = 100% of fluorescence before bleaching).

We further investigated the nature of the filaments by FRAP (fluorescence recovery after photobleaching) experiments, which showed that the BsYFP-MreB filaments had a rapid turnover (Fig. 3A) with a half-time recovery of 12 s on average (Fig. 4A), independent of filament thickness. Interestingly, recovery did not reach more than 50% of initial levels (and half time recovery refers to these 50%) (Fig. 4A). To study this effect further, we performed re-FRAPing of previously bleached and recovered areas. Intriguingly, recovery was again only 50% of initial fluorescence levels (Fig. 4A), indicating that MreB filaments consist of two different populations, an exchangeable and a non- (or very slowly) exchangeable fraction. This could be due to several reasons. Firstly, we have shown that at least 50% of all MreB molecules are tightly associated with the polymerized fraction (see above). Thus, at least half of the MreB molecules are within filamentous structures and are not available for exchange. Secondly, MreB clearly has membrane affinity, which was recently also shown for *E. coli* and *Thermotoga* MreB [19], so MreB molecules have limited diffusion within the S2 cell cytosol and along the membrane. Thirdly, we show that MreB filaments clearly consist of bundles of filaments, and filaments within the bundle are likely to be more stable than filaments having less lateral contacts to other protofilaments. MreB recovery has been reported to be 2.5 min for YFP-MreB in *B. subtilis* cells [10]. However, it must be taken into account that cellular parameters may be quite different between bacteria and Schneider cells, in terms of ion concentrations, crowding conditions, abundance of membranes, and cellular level of MreB, all of which might be critical parameters in its polymerization. In any event, dynamic remodelling of MreB filaments appears to be intrinsic to the protein. An extension of YFP-MreB filaments could readily be observed between 3 min intervals (Fig. 3A), and even between 1 min intervals (data not shown), revealing that MreB also dynamically polymerizes in a heterologous cell system. When a mutant allele of MreB in which ATPase activity is supposedly affected (D158A) is expressed in S2 cells, highly elongated filaments with extensive fluorescence were observed (Fig. 3B, movie S3). As expected, recovery after photobleaching of mutant YFP-MreB was much slower than that of wild type YFP-MreB (Fig. 3C). Only 5% fluorescence was recovered after 4 min, whereas 50% of wild type fluorescence was recovered within this time frame (Fig. 4C). Maximal recovery was 20% fluorescence after 40 min (data not shown), and thus average half time recovery was 20 min. These experiments verify that the D158A mutation strongly reduces MreB filament dynamics *in vivo*. Interestingly, mutant MreB filaments initially assembled at the cell membrane (Fig. 3D and movie S3), and then became detached after extended incubation of cells (Fig. 3E and F, movie S4). When YFP-MreBD158A was expressed and S2 cells continued to grow, YFP-MreB filaments were detectable in cells that had undergone cell division (data not shown), showing that MreB structures are very stable and last into the next cell generation. Occasionally, cells were observed to be stuck in cell division, being unable to constrict through the MreB filaments, showing that their high rigidity can resist cytokinesis (Fig. 3E and F).

Interestingly, prolonged expression of ATPase mutant MreB (i.e. for more than 4 h) resulted in the formation of membrane extrusions surrounding fluorescent YFP-MreB filaments (Fig. 3G, 3H and movie S5). These observations suggest that MreB polymerization can exert force against the membrane, in analogy to actin, which can push membranes [20], [21], and reveal yet another conserved feature between actin and MreB.

We also addressed the question if MreB filaments are affected by the availability of ATP. Cells were treated with carbonyl cyanide p-trifluoromethoxyphenylhydrazone (FCCP), which uncouples oxidative phosphorylation, leading to a rapid drop in cellular ATP levels [22], [23]. The caveat of these experiments is the fact that cells ultimately die in response to ATP depletion. However, clear signs of apoptosis (e.g. formation of phase bright vesicles at the membrane, shrinkage/disruption of the nucleus) were not seen before 90 min after addition of FCCP, and most cells did not show any signs for several hours. Interestingly, YFP-MreB filaments were no longer seen 10 to 20 min after addition of FCCP, but only cytosolic accumulations showing fluorescence (Fig. 1H, I). 90 min after addition of FCCP, YFP-MreB was dispersed throughout the cytosol (Fig. 1J), but not degraded (data not shown). These data suggest that MreB filaments rapidly disintegrate in response to lowered ATP levels, at a time point when cells do not show any signs of damage.

### MreB paralogs form a single filamentous structure underneath the cell membrane

**Figure 5:**
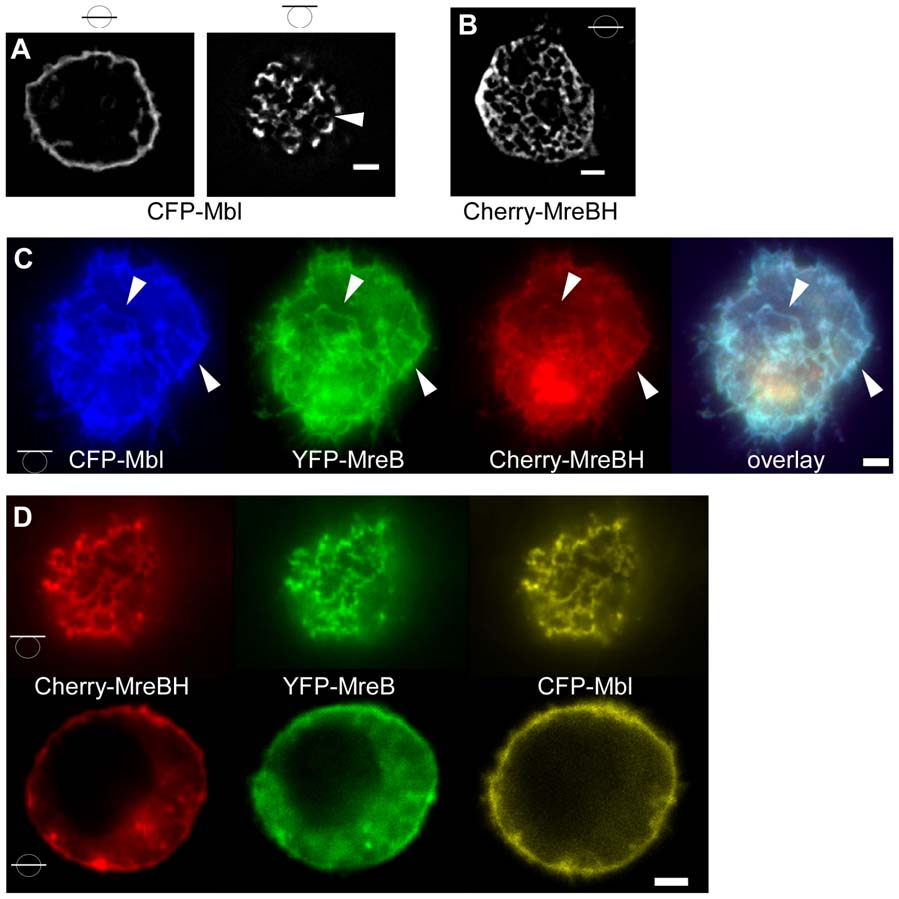
Expression of MreB paralogs in S2 cells. A) 3D deconvoluted images of CFP-Mbl, B) 3D deconvoluted image of mCherry-MreBH (middle plane). Bars in circles indicate position of image planes. C) Top view of a cell expressing YFP-MreB, CFP-Mbl and mCherry-MreBH, triangles indicate identical structures. D) Top and middle plane of a cell expressing all three MreB paralogs. White bars 2 µm.

Extending our analysis to the MreB paralogs Mbl and MreBH, we found that upon individual expression in S2 cells, both proteins formed filamentous structures, however CFP-Mbl filaments were exclusively localized at the cell membrane (Fig. 5A), like those of MreB, while MreBH filaments were also present within the cytosol away from the cell membrane, and were not visibly accumulated at the cell membrane (Fig. 5B). MreBH filaments formed a network-like structure, whose individual filaments did not show curvature like MreB or Mbl filaments, but appeared to be straight. However, we can not exclude that the network is composed of very short curved filaments that are not resolved by fluorescence microscopy. The network extended throughout the cells, and was not visibly accumulated at specific subcellular regions, suggesting that MreBH did not accumulate at the endoplysmatic reticulum or Golgi membrane, although we can not exclude this possibility. Unlike MreB, Mbl filaments were invariably curved, and occasionally had a helical architecture (Fig. 5A). These results show that MreB and Mbl have intrinsic membrane affinity, while MreBH filaments do not associate with the cell membrane by itself (note that the three proteins display only 55% sequence identity). Like MreB, Mbl and MreBH filaments showed high turnover as determined by FRAP analyses, and recovered within few minutes (Fig. 4B and data not shown). Mbl recovery reached 50%, and therefore, half time recovery was 240 s, compared with 12 s for MreB (Fig. 4A). After 12 s, only 8% of Mbl fluorescence was recovered, in contrast to 25% of MreB fluorescence, showing that Mbl has a slower turnover rate. Mbl filaments in *B. subtilis* cells also show turnover in a frame of 5 minutes [14], revealing that filament dynamics are an intrinsic property of all three MreB paralogs.

Intriguingly, when all three proteins were co-expressed, each carrying a different FP fusion, they co-polymerized underneath the cell membrane (Fig. 5C and 5D), showing that a) they form mixed polymers or co-polymers, and b) MreB/Mbl filaments can recruit MreBH filaments to the cell membrane. Therefore, membrane-association of MreB and of Mbl is sufficient to also attach MreBH to the membrane. Filaments were generally curved and much longer than those formed by MreB or Mbl themselves, revealing that the mixed polymers of the MreB paralogs obtain a different architecture compared with the single polymers.

### Mbl can recruit the RodZ ortholog to the cell membrane

**Figure 6:**
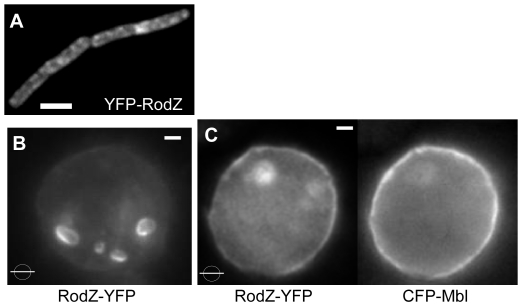
Recruitment of proteins to Mbl-membrane structures. A) Localization of YFP-RodZ in *Bacillus subtilis* cells. B) Membrane protein RodZ-YFP expressed in S2 cells by itself, or C) together with CFP-Mbl. White bars 2 µm.

We also investigated the localization of an interaction partner for MreB, membrane protein RodZ [24], which was shown to influence MreB localization in two Gram negative bacterial species [17], [25], [26]. RodZ has a single membrane span, and its cytosolic part may anchor MreB to the cell membrane. RodZ localizes to irregular sites within the *B. subtilis* cell membrane (Fig. 6A), like its counterparts in proteobacteria [27]. When expressed by itself in S2 cells, RodZ from *B. subtilis* localized to internal membrane compartments (Fig. 6B), most likely because eukaryotic membrane-targeting sequences are missing in the bacterial protein. However, when co-expressed with Mbl, RodZ-YFP fluorescence was mostly present underneath the cell membrane, and less so at internal membrane systems (6C). This reveals that Mbl and RodZ functionally interact, and that Mbl has the striking capacity to relocalize a protein from internal membranes to the cell membrane in a heterologous cell system. It is possible that RodZ normally reaches the plasma membrane, but is recycled in the absence of Mbl, or that its movement to the plasma membrane is facilitated by interactions with Mbl. Presently, we can not distinguish between a trapping mechanism or a more active targeting mechanism. In toto, these data reinforce the idea that *B. subtilis* MreB paralogs do not need a dedicated membrane anchor, but are able to direct the localization of a membrane protein within the cell membrane.

### Conclusions

Taken together, our data show that bacterial MreB proteins from *B. subtilis* self-organize as dynamic filamentous structures underneath the cell membrane in a heterologous cell system. These proteins also form mixed polymers in *E. coli* cells [28], colocalize and interact in *B. subtilis* cells [10], revealing that co-assembly is independent of the cellular background. MreB from *B. subtilis* and from *E. coli* clearly and readily form bundles of filaments, and BsMreB filaments are highly dynamic, revealing that filament turnover is also an intrinsic property of this bacterial actin ortholog, and that bacterial MreB proteins do not need specific nucleators to efficiently form filaments. Moreover, MreB filaments were able to distort the cell membrane, indicating that they can exert force onto the membrane and may also contribute mechanical strength to the bacterial cell, for which evidence has recently been put forward [29]. MreB and Mbl filaments can recruit cytosolic as well as internal membrane proteins to the cell membrane, revealing that they may be ideally suited for applications in synthetic biology.

## Methods

### Schneider cell culture and transient transfection

*D. melanogaster* S2 Schneider cells were grown in Schneider's Drosophila medium (Lonza Group Ltd.) supplemented with 5–10% (v/v) fetal calf serum (FCS) at 25°C without addition of CO_2_. Cells were passaged every 2 to 3 days to maintain optimal growth. S2 cells were transfected using the cationic lipid Cellfectin (Invitrogen). The S2 cells were spread in a 6–well plate at 1×10^6^ per well in 3 ml medium containing 5% FCS. Supercoiled plasmids (0.3 µg of each plasmid) were complexed with lipid (10 µl Cellfectin reagent) in 200 µl serum-free medium. The complex was incubated at room temperature for 15 min, filled up with serum-free medium to 1 ml and then added to cells from which the growth medium was removed (cells were washed 1 X with serum-free medium). After 18 hrs, the complex suspension was removed and replaced by 3 ml of medium containing 10% FCS. After further incubation for 24 h, the production of the proteins was induced by adding CuSO_4_ to a final concentration of 1 mM.

### Immunofluorescence

100 µl of transfected cells were transferred onto a poly-L-lysine treated glass slide and left to settle for 15 min at 37°C. Medium was removed and cells fixed with 100 µl fixation buffer [1x PBS, 0.1% triton X100, 3% formaldehyde (methanol free)] for 10 min at RT. After washing twice with washing buffer (1x PBS, 0.1% triton×100) for 5 minutes at RT, 100 µl image-iT Fx signal enhancer (Invitrogen) were applied and incubated over night at 4°C. For visualisation of tubulin, α-tubulin antibodies (1:10 dilution in 1xPBS, 0.1% triton×100, 1% non fat milk, origin: mouse) were added and incubated for 90 min at RT. Unbound antibodies were removed by three times washing with washing buffer (5 min, RT) and tubulin signals were visualized by the secondary antibody goat anti mouse coupled to alexa fluor 555 (Invitrogen) (1:100 dilution in 1x PBS, 0.1% triton×100, 1% non fat milk 1 h, RT). After washing for 5 min at RT, cells were counterstained with DAPI (5 µg/ml in washing buffer, 5 min, RT) and mounted with fluorescent mounting medium (Dako).

For visualization of actin, FITC labelled phalloidin was added (2 µg/ml in washing buffer, 2 h, RT). The sample was washed three times with washing buffer (5 min, RT), subsequently cells were counterstained with DAPI (5 µg/ml in washing buffer, 5 min, RT) and mounted with fluorescent mounting medium (Dako).

### Plasmids

To obtain C-terminal fluorescent protein fusions the plasmid pFD1 was constructed by combining the multiple cloning site as well as the coding sequence of the fluorophore of the plasmid pSG1164 [30] with the plasmid pRmHa3 using *Kpn*I and *Spe*I. [15]

As *B. subtilis* strains carrying a fluorescent version of MreB, MreBD158A MreBH and Mbl were available (table 1), the coding region was amplified using chromosomal DNA of the respective strain and the oligonucleotides listed in table 2. Products were cloned into pFD1 using the restriction endonucleases described in table 2 to obtain the plasmids listed in Table 3. EcMreB-RFP^SW^ (Bendezu *et al.*, 2009) was amplified using chromosomal DNA of strain FD76 and oligonucleotides EcMreB-RFPSWup, EcMreB-RFPSWdown. The resulting fragment was fused into pFD1 using *Kpn*I and *BamH*I. RodZ was amplified using the oligonucleotides RodZup RodZdown and chromosomal DNA of *B. subtilis* PY79. The resulting fragment was integrated into pFD1 using *Apa*I and *Cla*I. For transfection all plasmids were adjusted to a concentration of 0.1 µg/µl.

**Table 1:** Strains.

Name	Organism	Genotype	Ref.
JS36	*B. subtilis* PY79	*Pxyl-yfp-mreB::amy*	[31]
JS40	*B. subtilis* PY79	*Pxyl-mCer-mbl*	[31]
JS99	*B. subtilis* PY79	*Pxyl-mCherry-MreBH*	This work
PY79	*B. subtilis*	Wild type *Bacillus subtilis* subsp. *Subtilis*	
JS51	*B. subtilis* PY79	*Pxyl-gfp-mreBD158A::amy*	[10]
FB76	*E. coli*	*mreB’-rfp-‘mreB yhdE<>cat*	[17]
FD305	*B. subtilis* PY79	*Pxyl-yfp-rodZ::amy*	This work

**Table 2:** Oligonucleotides.

Name	Sequence	Restriction site
EcMreB-RFP SW up	TCAGGTACCATGTTGAAAAAATTTCGTGGCAT	KpnI
EcMreB-RFP SW down	TCAGGATCCTTACTCTTCGCTGAACAGGTC	BamHI
BsMreBup	ACTGGTACCATGAGTAAAGGAGAAGAACTTTTC	*Kpn*I
BsMreBdown	TCAACTAGTTTATCTAGTTTTCCCTTTGAAAAG	*Spe*I
BsMreBHup	ATCGGTACCATGGTGAGCAAGGGCGAG	*Kpn*I
BsMreBHdown	ATCACTAGTCTATTTAATTGCCTTTTGCAG	*Spe*I
BsMblup	ACTGGTACCCTGCAGATGGTTTCAAAAGGCGAAG	*Kpn*I
BsMbldown	ATCACTAGTTCAGCTTAGTTTGCGTTTAG	*Spe*I
RodZup	TCAGGGCCCATGTCATTGGATGATCTCCAAG	*Apa*I
RodZdown	TCAATCGATCCCGCCAATCCAGGAGCTGTCTGATGC	*Cla*I
MreBD158Aup	ACTGGTACCATGAGTAAAGGAGAAGAACTTTTC	*Kpn*I
MreBD158Adown	TCAACTAGTTTATCTAGTTTTCCCTTTGAAAAG	*Spe*I
RodZdown2	TCAATCGATTCAAATCCAGGAGCTGTCTGATGC	*Cla*I

**Table 3:** Generated Plasmids.

Name	
pFD1	pRmHa3 combined with the MCS of pSG1164 and *yfp*
pFD2	constructed as pFD1. *Yfp* replaced by cer*cfp.*
pCR1 (*bla yfp-mreB*)	yfp-mreB in pFD1
pCR2 (*bla, BsCFP-mbl*)	BsCFP-mbl in pFD1
pCR3 (*bla, mCherry-mreBH*)	mCherry-mreBH in pFD1
pFD3 (*bla, yfp-mreB D158A*)	yfp-mreB D158A in pFD1
pDD1 (*bla, EcMreB-RFP SW*)	EcMreB-RFP SW in pFD1
pFD4(*bla, rodZ-yfp*)	rodZ-yfp in pFD2
pFD5 (*bla,cat, yfp-rodZ*)	yfp-rodZ in psG1729

To obtain an N-terminal fluorescent protein fusion of RodZ (YmfM) the coding region was amplified using the oligonucleotides RodZup, RodZdown2 and chromosomal DNA of *B. subtilis*. The resulting fragment was cloned into psG1729 [30] using *Apa*I and *Cla*I to obtain pFD9.

### Sedimentation assays

S2 cells were suspended in homogenization buffer (10 mM HEPES-KOH pH 7.4, 150 mM KCl, 0.5 mM MgSO_4_, 1 mM DTT, 1× NEB protease inhibitor cocktail, 4°C) and homogenized by passage of the cells through a needle (diameter 0.4 mm) 20 times. The homogenate was cleared by centrifugation at 800 g for 5 minutes. To sediment polymerized MreB the sample was centrifuged for 60 min at 18 000 g (RT). The pellet was resuspended in an equal volume of homogenization buffer. The proteins were separated by SDS-PAGE and analyzed by western blot using anti GFP and anti MreB antibodies (dilution 1:500). Secondary HRP conjugated anti rabbit antibodies (NEB) were employed in a dilution of 1: 1000.

### Image acquisition

Fluorescence microscopy was performed using a Zeiss Observer Z1 equipped with a 1.45 NA objective and a Photometrix Cascade CCD camera. The fluorophores were exited by exposition to a laser of 488 nm wave length, or 561 nm for RFP. CFP fluorescence was observed using a HG/Xenon light source and a filter set corresponding to the excitation and emission wavelength of CFP. FRAP experiments were performed using a laser of 405 nm wave length. The membrane was visualized with FM4-64 (final concentration 2.5 µg/ml). Images were processed with the Metamorph 7.5.5 software. 3D-deconvolution was performed applying the Autodeblur X1.4.1 (AutoQuant Imaging) algorithm.

## Supporting Information

Movie S1 **3D deconvoluted stack of YFP-MreB filaments expressed in an S2 Schneider cell.** Movie 5 frames/s.
(AVI)

Movie S2 **Stack of EcMreB-RFP^SW^ filaments in an S2 Schneider cell, taken from the middle of the cell to the surface.** Movie 5 frames/s.
(AVI)

Movie S3 **3D reconstruction of an S2 cell expressing YFP-MreB-D158A mutant filaments.** Movie 5 frames/s.
(AVI)

Movie S4 **3D deconvoluted stack of YFP-MreB-D158A filaments expressed in an S2 Schneider cell, taken from top to bottom.** Movie 5 frames/s.
(AVI)

Movie S5 **3D deconvoluted stack of YFP-MreB-D158A filaments expressed in an S2 Schneider cell, from which MreB has generated membrane protrusions, taken from top to bottom.** Movie 5 frames/s.
(AVI)
